# Di­aqua­bis­(nicotinamide-κ*N* ^1^)bis­(thiocyanato-κ*S*)cobalt(II)

**DOI:** 10.1107/S1600536814011453

**Published:** 2014-05-31

**Authors:** Deepanjali Pandey, Shahid S. Narvi, Siddhartha Chaudhuri

**Affiliations:** a Department of Chemistry, Motilal Nehru National Institute of Technology, Allahabad 211 004, India; b Bose Institute, Kolkata 700 009, West Bengal, India

## Abstract

In the title compound, [Co(NCS)_2_(C_6_H_6_N_2_O)_2_(H_2_O)_2_], the Co^II^ cation is located on an inversion centre and is coordinated by two thio­cyanate anions, two nicotinamide mol­ecules and two water mol­ecules in a distorted N_2_O_2_S_2_ octa­hedral geometry. The amide group is twisted by 31.30 (16)° with respect to the pyridine ring. In the crystal, mol­ecules are linked by O—H⋯O, O—H⋯S and N—H⋯S hydrogen bonds into a three-dimensional supra­molecular network. Weak π–π stacking is observed between parallel pyridine rings of adjacent mol­ecules, the centroid–centroid distance being 3.8270 (19) Å.

## Related literature   

For general background and applications of transition-metal complexes with biochemically active ligands, see: Antolini *et al.* (1982 ▶); Krishnamachari (1974 ▶). For related structures, see: Hökelek *et al.* (2009*a* ▶,*b* ▶); Özbek *et al.* (2009 ▶).
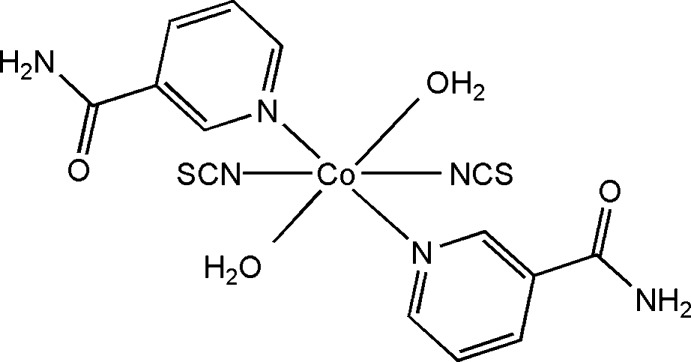


## Experimental   

### 

#### Crystal data  

[Co(NCS)_2_(C_6_H_6_N_2_O)_2_(H_2_O)_2_]
*M* *_r_* = 455.38
Triclinic, 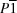

*a* = 7.5475 (19) Å
*b* = 8.054 (2) Å
*c* = 8.932 (2) Å
α = 73.347 (4)°
β = 70.067 (4)°
γ = 66.559 (4)°
*V* = 461.07 (19) Å^3^
*Z* = 1
Mo *K*α radiation
μ = 1.19 mm^−1^
*T* = 100 K
0.35 × 0.33 × 0.31 mm

#### Data collection  

Bruker SMART CCD area-detector diffractometer
Absorption correction: multi-scan (*SADABS*; Bruker, 2002 ▶) *T*_min_ = 0.68, *T*_max_ = 0.71
2450 measured reflections
1626 independent reflections
1469 reflections with *I* > 2σ(*I*)
*R*_int_ = 0.017

#### Refinement  

*R*[*F* ^2^ > 2σ(*F* ^2^)] = 0.036
*wR*(*F* ^2^) = 0.097
*S* = 1.07
1626 reflections
130 parameters
2 restraints
H atoms treated by a mixture of independent and constrained refinement
Δρ_max_ = 0.60 e Å^−3^
Δρ_min_ = −0.39 e Å^−3^

### 

Data collection: *SMART* (Bruker, 2007 ▶); cell refinement: *SAINT* (Bruker, 2007 ▶); data reduction: *SAINT*; program(s) used to solve structure: *SHELXTL* (Sheldrick, 2008 ▶); program(s) used to refine structure: *SHELXTL*; molecular graphics: *SHELXTL*; software used to prepare material for publication: *SHELXTL*.

## Supplementary Material

Crystal structure: contains datablock(s) global, I. DOI: 10.1107/S1600536814011453/xu5790sup1.cif

Structure factors: contains datablock(s) I. DOI: 10.1107/S1600536814011453/xu5790Isup2.hkl

CCDC reference: 850080

Additional supporting information:  crystallographic information; 3D view; checkCIF report

## Figures and Tables

**Table 1:** Selected bond lengths (Å)

Co—N1	2.050 (2)
Co—N2	2.119 (2)
Co—O2	2.0724 (18)

**Table 2:** Hydrogen-bond geometry (Å, °)

*D*—H⋯*A*	*D*—H	H⋯*A*	*D*⋯*A*	*D*—H⋯*A*
O2—H2*A*⋯O1^i^	0.83 (2)	1.89 (2)	2.690 (2)	161 (4)
O2—H2*B*⋯S1^ii^	0.82 (4)	2.41 (3)	3.204 (3)	163 (3)
N3—H3*A*⋯S1^iii^	0.86	2.66	3.425 (3)	149
N3—H3*B*⋯S1^iv^	0.86	2.63	3.422 (3)	153

Symmetry codes: (i) 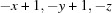; (ii) 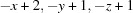; (iii) 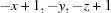; (iv) 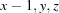.

**Fig. 1.**
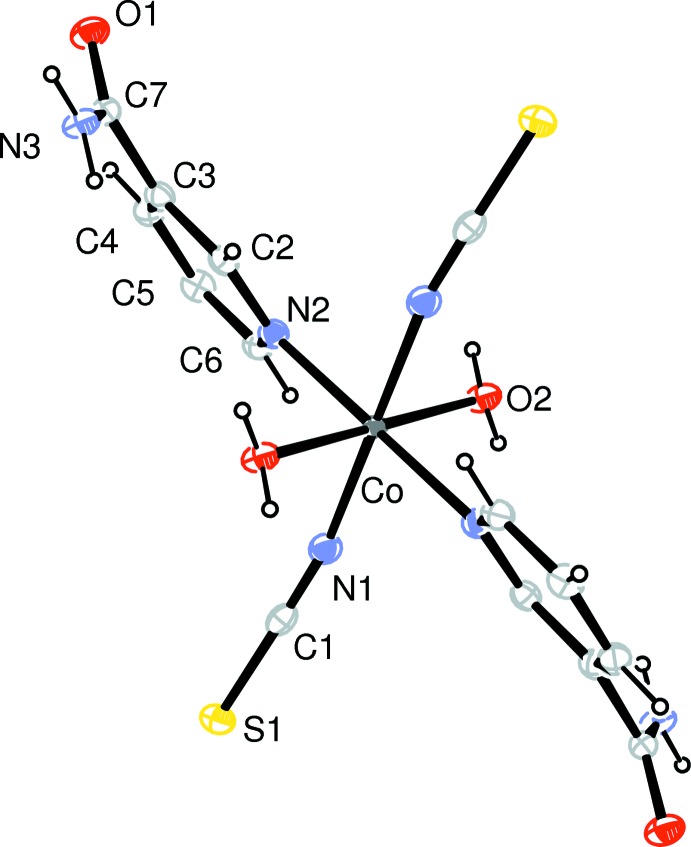
ORTEP view of complex[Co(nicotinamide)2(thiocynate)2(H2O)2] with atom labelling.

**Fig. 2.**
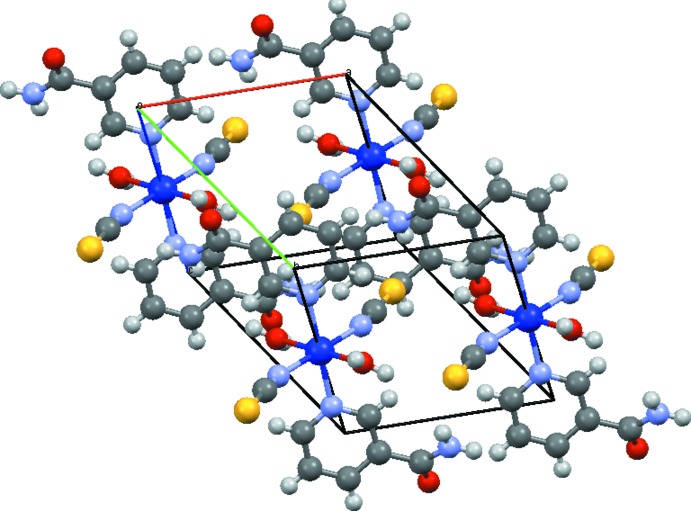
Packing diagram of the Complex. Hydrogen bonds are shown as dashed lines.

## supplementary crystallographic information

### 1. Comment

The structural unit of hydrogen bonded framework of cobalt is depicted in Figure 1. Co(II) is at a slightly distorted octahedral coordination environment. The equatorial positions are occupied by two nitrogen atoms from two nicotinamide ligands, the Co *N*(nicotinamide) bond length is 2.117 (3) angstrom, and two oxygen atoms from two water molecules, the Co O(water) bond length is 2.075 (2)angstrom and two nitrogen atoms from NCS groups occupy the axial positions with bond length of 2.049 (3)angstrom for Co *N*(thiocyanate). The O(water) Co O(water), *N*(thiocyanate) Co *N*(thiocyanate), and *N*(nicotinamide) Co *N*(nicotinamide) angles are constrained by symmetry to 180. The *N*(thiocyanate) Co *N*(nicotinamide), O(water) Co *N*(thiocyanate), and O(water) Co *N*(nicotinamide) angles are 87.48, 91.9, and 90.50 respectively, indicating a slightly distorted octahedral coordination for the Co ion. Both ligands generally acts as bidentate, however in this polymer plays as unidentate. The thiocynate SCN presents multiform coordination modes connecting the metal ions with terminal and or bridging fashions. According to the concept of hard soft acid base the SCN ion prefers to bind to Cd(II) centre in both N and S bonded fashion, whereas in the only N terminal mode to Co(II) ion·As expected, the SCN anion is almost linear angle: 178 and coordinates in a little bent fashion to Co, exhibiting a Co—N—C angle of 159.56. These structural features have already been observed in other thiocyanato-containing metal complexes. As a result of the *trans* orientation of two terminal N-bonded thiocyanate groups around the Co(II) atom, the bond angle N(1) Co(1) N(1) is 180. The S C and C N distances of 1.638 (2) angtrom and 1.158 (2)angstrom in the SCN– moiety show the normal structure of the thiocyanate in the complex which is also observed in other thiocyanate complexes·The nicotinamides molecules are *trans* to each other with angle N(2) Co N(2) is 180. The nicotinamide ligand generally acts as a bidentate chelating ligand, coordinating to the metal ion through the carbonyl O and pyridine N atoms, but in this structure it acts as a unidentate ligand in which the pyridine N is coordinated to the Co ion while the carbonyl O is involved in hydrogen bonding with another water molecule·Water used as solvent whereas it involved in coordination with metal ions and act as ligand. The coordination of nitrogen atoms of each thiocynate molecules and nicotinamide molecules results in the formation of two symmetrical axis N1–Co–N1, N2–Co–N2 respectively. The oxygen atoms of water molecules describe the third axis O(1) W A–Co—O(1)WB. The Co Co distance spaced by the thiocynate ligand is 7.548 Å. The discrete units are connected by bifurcated hydrogen bonds between the coordinated water molecules and terminal thiocynate sulfur atoms forms O(2)—H(2) W A—-S(1) interlayer hydrogen bonding gives one-dimensional chain and forming ladder like structure. Further Oxygen atom of amide group from nicotinamide molecule makes hydrogen bonding with hydrogen atom of water molecule C(7)- - O(1)—H(2)WB to afford a 2-D layered architecture. As can be seen from the packing diagram, the Co atoms are located at the centre of the axis of the unit cell and the molecules of polymer are linked by intermolecular hydrogen bond O–H—O, O–H—-S and N–H—-S hydrogen bonds, forming a supramolecular structure. Dipole dipole and van der Waals interactions are also effective in the molecular packing. Remarkably, each sulfur atom of thiocynate molecule plays a trifurcated role to be involved in the hydrogen bonding with one hydrogen atom from water molecules and two hydrogen atoms from nicotinamide molecules thus formation of three S(1)—H2WA, S(1)—H3A andS(1)—H3B interlayer respectively. With the aid of these contacts polymer affords three-dimensional structure.

### 2. Experimental

An aqueous solution (10 ml) of Cobalt nitrate (0.2460 g, 1 mmol) and Potassium thiocyanate (0.196 g, 2 mmol) was slowly added drop wise to hot aqueous solution (10 ml) of Nicotinamide (0.241 g, 2 mmol) with stirring. Greenish blue colour solution was obtained. After filtration the final clear solution left undisturbed at room temperature for slow evaporation. Next day, needle shaped greenish blue crystals were collected and dried *in vacuo* over silica gel. Crystals suitable for single crystals X-ray diffraction was manually selected and immersed in silicon oil.

### 3. Refinement

Water H atoms were located in a different Fourier map and refined in riding mode with distance constraint of O—H = 0.82 (2) Å, U_iso_(H) = 1.5U_eq_(O). Other H atoms were placed in calculated positions with C—H = 0.93 and N—H = 0.86 Å, and refined in riding mode with U_iso_(H) = 1.2U_eq_(N,C).

### Crystal data

**Table:**

[Co(NCS)_2_(C_6_H_6_N_2_O)_2_(H_2_O)_2_]	*Z* = 1
*M**_r_* = 455.38	*F*(000) = 233
Triclinic, *P*1	*D*_x_ = 1.640 Mg m^−^^3^
Hall symbol: -P 1	Mo *K*α radiation, λ = 0.71073 Å
*a* = 7.5475 (19) Å	Cell parameters from 1626 reflections
*b* = 8.054 (2) Å	θ = 2.5–25.2°
*c* = 8.932 (2) Å	µ = 1.19 mm^−^^1^
α = 73.347 (4)°	*T* = 100 K
β = 70.067 (4)°	Prism, pink
γ = 66.559 (4)°	0.35 × 0.33 × 0.31 mm
*V* = 461.07 (19) Å^3^	

### Data collection

**Table:**

Bruker SMART CCD area-detector diffractometer	1626 independent reflections
Radiation source: fine-focus sealed tube	1469 reflections with *I* > 2σ(*I*)
Graphite monochromator	*R*_int_ = 0.017
phi and ω scans	θ_max_ = 25.2°, θ_min_ = 2.5°
Absorption correction: multi-scan (*SADABS*; Bruker, 2002)	*h* = −9→8
*T*_min_ = 0.68, *T*_max_ = 0.71	*k* = −8→9
2450 measured reflections	*l* = −6→10

### Refinement

**Table:**

Refinement on *F*^2^	Primary atom site location: structure-invariant direct methods
Least-squares matrix: full	Secondary atom site location: difference Fourier map
*R*[*F*^2^ > 2σ(*F*^2^)] = 0.036	Hydrogen site location: inferred from neighbouring sites
*wR*(*F*^2^) = 0.097	H atoms treated by a mixture of independent and constrained refinement
*S* = 1.07	*w* = 1/[σ^2^(*F*_o_^2^) + (0.0582*P*)^2^ + 0.1481*P*] where *P* = (*F*_o_^2^ + 2*F*_c_^2^)/3
1626 reflections	(Δ/σ)_max_ = 0.001
130 parameters	Δρ_max_ = 0.60 e Å^−^^3^
2 restraints	Δρ_min_ = −0.39 e Å^−^^3^

### Special details

**Table:**

Geometry. All esds (except the esd in the dihedral angle between two l.s. planes) are estimated using the full covariance matrix. The cell esds are taken into account individually in the estimation of esds in distances, angles and torsion angles; correlations between esds in cell parameters are only used when they are defined by crystal symmetry. An approximate (isotropic) treatment of cell esds is used for estimating esds involving l.s. planes.
Refinement. Refinement of F^2^ against ALL reflections. The weighted R-factor wR and goodness of fit S are based on F^2^, conventional R-factors R are based on F, with F set to zero for negative F^2^. The threshold expression of F^2^ > 2sigma(F^2^) is used only for calculating R-factors(gt) etc. and is not relevant to the choice of reflections for refinement. R-factors based on F^2^ are statistically about twice as large as those based on F, and R- factors based on ALL data will be even larger.

### Fractional atomic coordinates and isotropic or equivalent isotropic displacement parameters (Å^2^)

**Table:**

	*x*	*y*	*z*	*U*_iso_*/*U*_eq_	
Co	0.5000	0.5000	0.5000	0.01075 (18)	
S1	1.10774 (10)	0.10828 (9)	0.64395 (8)	0.0188 (2)	
N1	0.7855 (3)	0.3745 (3)	0.5269 (3)	0.0190 (5)	
N2	0.5800 (3)	0.3846 (3)	0.2911 (3)	0.0156 (5)	
N3	0.2309 (3)	0.1182 (3)	0.2363 (3)	0.0183 (5)	
H3A	0.1408	0.0839	0.2280	0.022*	
H3B	0.2447	0.1099	0.3301	0.022*	
O1	0.3341 (3)	0.1985 (3)	−0.0328 (2)	0.0219 (4)	
O2	0.5635 (3)	0.7330 (3)	0.3582 (2)	0.0187 (4)	
C1	0.9198 (4)	0.2665 (4)	0.5745 (3)	0.0163 (6)	
C2	0.4546 (4)	0.3214 (3)	0.2666 (3)	0.0156 (5)	
H2	0.3342	0.3269	0.3443	0.019*	
C3	0.4961 (4)	0.2479 (4)	0.1301 (3)	0.0162 (6)	
C4	0.6758 (4)	0.2414 (4)	0.0138 (3)	0.0178 (6)	
H4	0.7067	0.1965	−0.0802	0.021*	
C5	0.8072 (4)	0.3027 (4)	0.0404 (3)	0.0188 (6)	
H5	0.9296	0.2971	−0.0344	0.023*	
C6	0.7542 (4)	0.3726 (4)	0.1800 (3)	0.0171 (6)	
H6	0.8438	0.4132	0.1973	0.021*	
C7	0.3480 (4)	0.1838 (3)	0.1049 (3)	0.0156 (6)	
H2A	0.572 (5)	0.748 (4)	0.260 (2)	0.023*	
H2B	0.662 (4)	0.750 (4)	0.363 (4)	0.023*	

### Atomic displacement parameters (Å^2^)

**Table:**

	*U*^11^	*U*^22^	*U*^33^	*U*^12^	*U*^13^	*U*^23^
Co	0.0119 (3)	0.0132 (3)	0.0084 (3)	−0.0052 (2)	−0.00355 (19)	−0.00102 (18)
S1	0.0173 (4)	0.0203 (4)	0.0188 (4)	−0.0074 (3)	−0.0069 (3)	0.0011 (3)
N1	0.0212 (13)	0.0203 (12)	0.0166 (12)	−0.0074 (10)	−0.0059 (10)	−0.0032 (9)
N2	0.0170 (12)	0.0159 (12)	0.0140 (11)	−0.0062 (9)	−0.0046 (9)	−0.0009 (9)
N3	0.0205 (12)	0.0254 (13)	0.0140 (11)	−0.0128 (10)	−0.0051 (9)	−0.0025 (9)
O1	0.0280 (11)	0.0270 (11)	0.0156 (10)	−0.0134 (9)	−0.0079 (8)	−0.0021 (8)
O2	0.0217 (10)	0.0247 (11)	0.0143 (9)	−0.0135 (9)	−0.0058 (8)	−0.0005 (8)
C1	0.0184 (14)	0.0207 (14)	0.0120 (13)	−0.0101 (12)	0.0003 (11)	−0.0060 (11)
C2	0.0159 (13)	0.0147 (13)	0.0140 (12)	−0.0044 (11)	−0.0040 (10)	−0.0001 (10)
C3	0.0185 (14)	0.0147 (13)	0.0141 (13)	−0.0053 (11)	−0.0052 (11)	0.0003 (10)
C4	0.0215 (14)	0.0182 (14)	0.0126 (13)	−0.0062 (11)	−0.0041 (11)	−0.0021 (10)
C5	0.0158 (14)	0.0215 (15)	0.0163 (13)	−0.0065 (11)	−0.0019 (11)	−0.0016 (11)
C6	0.0171 (14)	0.0175 (14)	0.0175 (14)	−0.0068 (11)	−0.0065 (11)	−0.0003 (11)
C7	0.0179 (14)	0.0140 (13)	0.0151 (13)	−0.0034 (11)	−0.0061 (11)	−0.0032 (10)

### Geometric parameters (Å, º)

**Table:**

Co—N1	2.050 (2)	O1—C7	1.237 (3)
Co—N1^i^	2.050 (2)	O2—H2A	0.838 (18)
Co—N2	2.119 (2)	O2—H2B	0.825 (18)
Co—N2^i^	2.119 (2)	C2—C3	1.394 (4)
Co—O2	2.0724 (18)	C2—H2	0.9300
Co—O2^i^	2.0724 (18)	C3—C4	1.391 (4)
S1—C1	1.652 (3)	C3—C7	1.505 (4)
N1—C1	1.160 (4)	C4—C5	1.378 (4)
N2—C6	1.337 (3)	C4—H4	0.9300
N2—C2	1.338 (3)	C5—C6	1.384 (4)
N3—C7	1.321 (3)	C5—H5	0.9300
N3—H3A	0.8600	C6—H6	0.9300
N3—H3B	0.8600		
			
N1—Co—N1^i^	180.0	Co—O2—H2A	116 (2)
N1—Co—O2	91.98 (8)	Co—O2—H2B	118 (2)
N1^i^—Co—O2	88.02 (8)	H2A—O2—H2B	106 (3)
N1—Co—O2^i^	88.02 (8)	N1—C1—S1	178.4 (2)
N1^i^—Co—O2^i^	91.98 (8)	N2—C2—C3	123.0 (2)
O2—Co—O2^i^	180.000 (1)	N2—C2—H2	118.5
N1—Co—N2	91.21 (9)	C3—C2—H2	118.5
N1^i^—Co—N2	88.79 (9)	C4—C3—C2	118.2 (2)
O2—Co—N2	90.66 (8)	C4—C3—C7	120.5 (2)
O2^i^—Co—N2	89.34 (8)	C2—C3—C7	121.3 (2)
N1—Co—N2^i^	88.79 (9)	C5—C4—C3	118.9 (2)
N1^i^—Co—N2^i^	91.21 (9)	C5—C4—H4	120.5
O2—Co—N2^i^	89.34 (8)	C3—C4—H4	120.5
O2^i^—Co—N2^i^	90.66 (8)	C4—C5—C6	119.0 (2)
N2—Co—N2^i^	180.0	C4—C5—H5	120.5
C1—N1—Co	159.3 (2)	C6—C5—H5	120.5
C6—N2—C2	117.8 (2)	N2—C6—C5	123.0 (2)
C6—N2—Co	122.02 (17)	N2—C6—H6	118.5
C2—N2—Co	120.17 (17)	C5—C6—H6	118.5
C7—N3—H3A	120.0	O1—C7—N3	122.5 (2)
C7—N3—H3B	120.0	O1—C7—C3	120.7 (2)
H3A—N3—H3B	120.0	N3—C7—C3	116.7 (2)

Symmetry code: (i) −*x*+1, −*y*+1, −*z*+1.

### Hydrogen-bond geometry (Å, º)

**Table:**

*D*—H···*A*	*D*—H	H···*A*	*D*···*A*	*D*—H···*A*
O2—H2*A*···O1^ii^	0.83 (2)	1.89 (2)	2.690 (2)	161 (4)
O2—H2*B*···S1^iii^	0.82 (4)	2.41 (3)	3.204 (3)	163 (3)
N3—H3*A*···S1^iv^	0.86	2.66	3.425 (3)	149
N3—H3*B*···S1^v^	0.86	2.63	3.422 (3)	153

Symmetry codes: (ii) −*x*+1, −*y*+1, −*z*; (iii) −*x*+2, −*y*+1, −*z*+1; (iv) −*x*+1, −*y*, −*z*+1; (v) *x*−1, *y*, *z*.

## References

[bb1] Antolini, L., Battaglia, L. P., Corradi, A. B., Marcotrigiano, G., Menabue, L., Pellacani, G. C. & Saladini, M. (1982). *Inorg. Chem.* **21**, 1391-1395.

[bb2] Bruker (2002). *SADABS* Bruker AXS Inc. Madison, Wisconsin, USA.

[bb3] Bruker (2007). *SMART* and *SAINT* Bruker AXS Inc. Madison, Wisconsin, USA.

[bb4] Hökelek, T., Dal, H., Tercan, B., Özbek, F. E. & Necefoğlu, H. (2009*a*). *Acta Cryst.* E**65**, m481–m482. 10.1107/S160053680901191X PMC2977549 21583735

[bb5] Hökelek, T., Yılmaz, F., Tercan, B., Özbek, F. E. & Necefoğlu, H. (2009*b*). *Acta Cryst.* E**65**, m768–m769. 10.1107/S1600536809021710 PMC2969345 21582699

[bb6] Krishnamachari, K. A. V. R. (1974). *Am. J. Clin. Nutr.* **27**, 108-111. 10.1093/ajcn/27.2.108 4812927

[bb7] Özbek, F. E., Tercan, B., Şahin, E., Necefoğlu, H. & Hökelek, T. (2009). *Acta Cryst.* E**65**, m341–m342. 10.1107/S1600536809006771 PMC2968619 21582108

[bb8] Sheldrick, G. M. (2008). *Acta Cryst.* A**64**, 112–122. 10.1107/S0108767307043930 18156677

